# The Cardiovascular Literature-Based Risk Algorithm (CALIBRA): Predicting Cardiovascular Events in Patients With Non-Dialysis Dependent Chronic Kidney Disease

**DOI:** 10.3389/fneph.2022.922251

**Published:** 2022-07-12

**Authors:** Luca Neri, Caterina Lonati, Jasmine Ion Titapiccolo, Jennifer Nadal, Heike Meiselbach, Matthias Schmid, Barbara Baerthlein, Ulrich Tschulena, Markus P. Schneider, Ulla T. Schultheiss, Carlo Barbieri, Christoph Moore, Sonia Steppan, Kai-Uwe Eckardt, Stefano Stuard, Francesco Bellocchio

**Affiliations:** ^1^ Clinical and Data Intelligence Systems-Advanced Analytics, Fresenius Medical Care Deutschland GmbH, Vaiano Cremasco, Italy; ^2^ Center for Preclinical Research, Fondazione IRCCS Ca’ Granda Ospedale Maggiore Policlinico, Milan, Italy; ^3^ Department of Medical Biometry, Informatics, and Epidemiology (IMBIE), Faculty of Medicine, University of Bonn, Bonn, Germany; ^4^ Department of Nephrology and Hypertension, Universitätsklinikum Erlangen, Friedrich-Alexander Universität Erlangen-Nürnber, Erlangen, Germany; ^5^ Medical Centre for Information and Communication Technology (MIK), University Hospital Erlangen, Erlangen, Germany; ^6^ Fresenius Medical Care, Deutschland GmbH, Bad Homburg, Germany; ^7^ Institute of Genetic Epidemiology, Faculty of Medicine and Medical Center - University of Freiburg, Freiburg, Germany; ^8^ Department of Medicine IV – Nephrology and Primary Care, Faculty of Medicine and Medical Center - University of Freiburg, Freiburg, Germany; ^9^ Department of Nephrology and Medical Intensive Care, Charité Universitätsmedizin Berlin, Berlin, Germany

**Keywords:** cardiovascular risk score, chronic kidney disease, cardiovascular events, hospitalization, machine learning, personalized medicine

## Abstract

**Background and Objectives:**

Cardiovascular (CV) disease is the main cause of morbidity and mortality in patients suffering from chronic kidney disease (CKD). Although it is widely recognized that CV risk assessment represents an essential prerequisite for clinical management, existing prognostic models appear not to be entirely adequate for CKD patients. We derived a literature-based, naïve-bayes model predicting the yearly risk of CV hospitalizations among patients suffering from CKD, referred as the CArdiovascular, LIterature-Based, Risk Algorithm (CALIBRA).

**Methods:**

CALIBRA incorporates 31 variables including traditional and CKD-specific risk factors. It was validated in two independent CKD populations: the FMC NephroCare cohort (European Clinical Database, EuCliD^®^) and the German Chronic Kidney Disease (GCKD) study prospective cohort. CALIBRA performance was evaluated by c-statistics and calibration charts. In addition, CALIBRA discrimination was compared with that of three validated tools currently used for CV prediction in CKD, namely the Framingham Heart Study (FHS) risk score, the atherosclerotic cardiovascular disease risk score (ASCVD), and the Individual Data Analysis of Antihypertensive Intervention Trials (INDANA) calculator. Superiority was defined as a ΔAUC>0.05.

**Results:**

CALIBRA showed good discrimination in both the EuCliD^®^ medical registry (AUC 0.79, 95%CI 0.76-0.81) and the GCKD cohort (AUC 0.73, 95%CI 0.70-0.76). CALIBRA demonstrated improved accuracy compared to the benchmark models in EuCliD^®^ (FHS: ΔAUC=-0.22, p<0.001; ASCVD: ΔAUC=-0.17, p<0.001; INDANA: ΔAUC=-0.14, p<0.001) and GCKD (FHS: ΔAUC=-0.16, p<0.001; ASCVD: ΔAUC=-0.12, p<0.001; INDANA: ΔAUC=-0.04, p<0.001) populations. Accuracy of the CALIBRA score was stable also for patients showing missing variables.

**Conclusion:**

CALIBRA provides accurate and robust stratification of CKD patients according to CV risk and allows score calculations with improved accuracy compared to established CV risk scores also in real-world clinical cohorts with considerable missingness rates. Our results support the generalizability of CALIBRA across different CKD populations and clinical settings.

## 1 Introduction

Patients with non-dialysis dependent chronic kidney disease (NDD-CKD) show higher cardiovascular morbidity and mortality relative to the general population (1, 2). The spectrum of cardiovascular disease (CVD) in CKD includes heart failure (HF), atrial fibrillation (AF), coronary artery disease (CAD), myocardial infarction (MI), cerebrovascular disease (CRVD), and peripheral arterial (occlusive) disease (PA(O)D) (3). About half of all NDD-CKD patients die subsequent to a cardiovascular event without progressing to kidney failure requiring renal replacement therapy (2, 4, 5). Given the significant burden of CVD in patients with NDD-CKD, cardiovascular risk stratification would enhance the effectiveness and efficiency of prevention programs (6).

Cardiovascular risk prediction in NDD-CKD is currently performed using predictive tools developed for the general population (3, 6, 7). However, the pathology and clinical manifestations of CVD differ in the presence of impaired kidney function (2, 3) and traditional risk factors sub-optimally predict CVD in CKD patients (3, 8, 9). The Kidney Disease: Improving Global Outcomes (KDIGO) clinical practice guidelines recommend using both estimated glomerular filtration rate (eGFR) and albuminuria for CVD risk assessment (7). Accordingly, A recent meta-analysis of the Chronic Kidney Disease Prognosis Consortium (CKD-PC) showed that inclusion of combined urinary albumin-to-creatinine ratio (ACR) and eGFR improves cardiovascular risk prediction in NDD-CKD populations (10). Of note, measures reflecting pathophysiological processes in the cardiovascular system, such as coronary artery calcium score or high-sensitivity cardiac troponin T (hs-cTnT), improved cardiovascular risk estimation in NDD-CKD (3, 11).

Given the lack of well-performing CVD risk stratification tools specifically addressing NDD-CKD patients, we derived a knowledge-based, Naïve-Bayes (NB) model predicting the yearly risk of cardiovascular hospitalizations in this patient group, namely the CArdiovascular, LIterature-Based, Risk Algorithm (CALIBRA). In the present study, we sought to evaluate the accuracy of CALIBRA in two independent cohorts of CKD patients, namely the FMC NephroCare cohort (European Clinical Database, EuCliD^®^) (12) and the German Chronic Kidney Disease (GCKD) study cohort (13). Moreover, we compared the performance of CALIBRA with that of three validated cardiovascular scores which are currently used for cardiovascular prediction in CKD patients.

## 2 Methods

### 2.1 Description of CALIBRA

CALIBRA is a literature-based naïve-bayes classifier (NBC) predicting the risk of at least one cardiovascular hospitalization within 1 year among NDD-CKD. An NBC is a simple probabilistic model based on application of the Bayes’ theorem. These tools are represented as directed acyclic graphs and their weights can be learned by data-driven algorithms or by codifying existing medical knowledge obtained from domain experts (14). NBCs can be exploited to generate metrics enabling broad prognostic reasoning. First, they generate a risk score representing the expected incidence of disease given a vector of known patients’ characteristics. Second, NBCs can be used to generate value of information (VOI) statistics and impact metrics. VOI statistics represent the reduction in uncertainty (i.e. entropy) in the outcome variable by measuring/observing the status of previously unknown variables (15). Therefore, it can be used to prioritize additional diagnostic testing or biomarker assays for patients with incomplete medical records. Conversely, impact metrics evaluates the magnitude of association of different subsets of evidence on the outcome variable (16). As a consequence, it can be used to estimate the potential impact of an intervention addressing some or all modifiable risk factors.

### 2.2 Derivation of CALIBRA

We derived a NB prognostic model entirely based on knowledge abstracted from medical literature.

**Figure 1:**
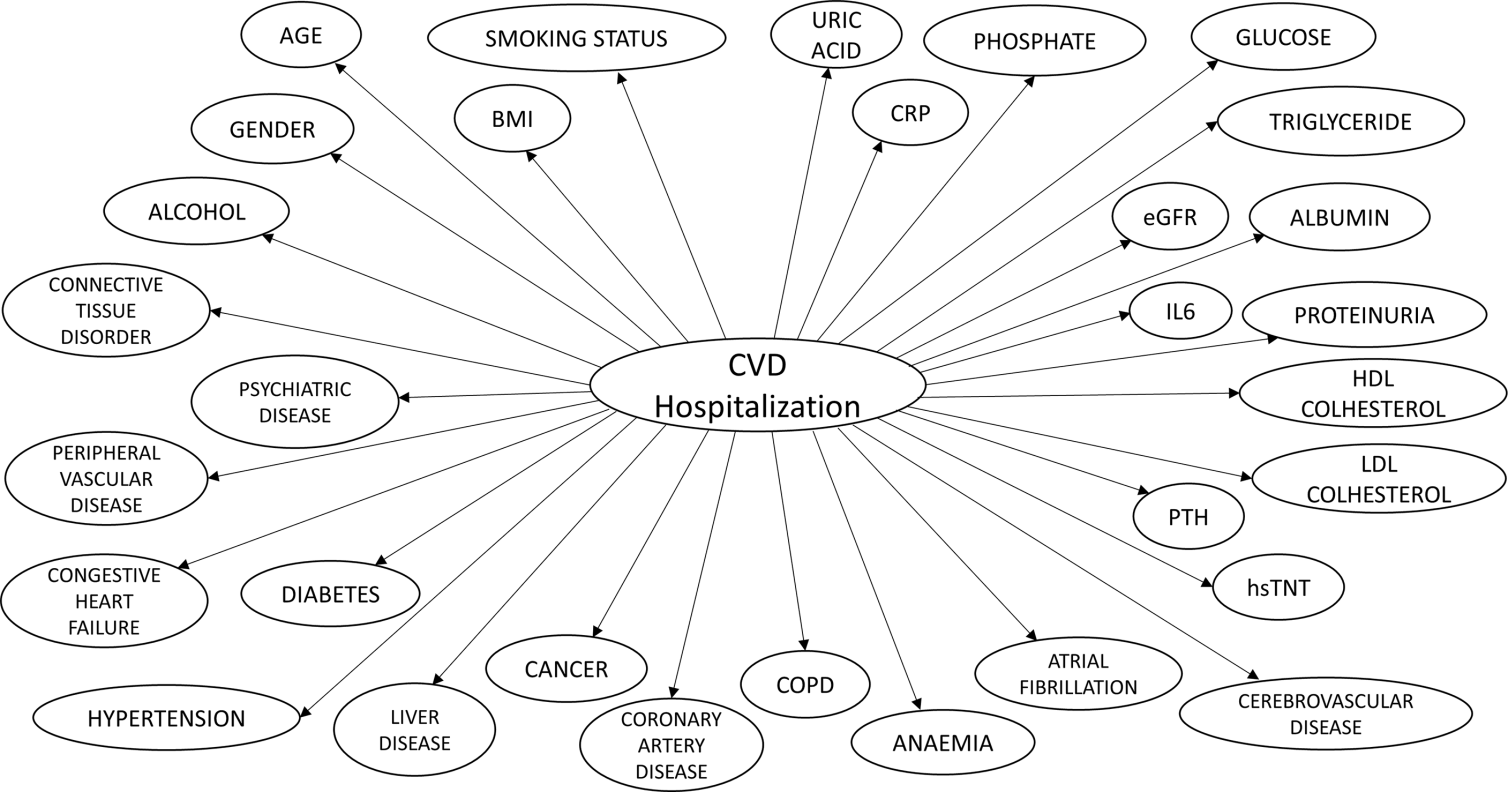
CALIBRA graphical structure.

CALIBRA graphical structure is shown in **Figure 1**. Model parameters feeding the conditional probability tables representing the relationship between each independent risk factor and outcomes were computed by abstracting and pooling effect size estimates from observational studies (**Supplementary Material**; **Appendix 1**). We used fixed effect meta-analysis using normalized study sample size as the weighting parameter. We included studies reporting data on fatal and non-fatal cardiovascular outcomes, whereas we excluded studies with follow up shorter than 1 year and those not reporting sufficient information for knowledge extraction. We also excluded researches focused on pediatric population, patients on renal replacement therapy or other disease populations. The process adopted to abstract medical knowledge from the literature and compute model parameters is reported in **Supplementary Material**; **Appendix 2**. We included in the final model 31 variables (**Table 1**). Details of model derivation have been described previously (17).

**Table 1:** Variables included in CALIBRA algorithm.

Group	Variable
**Demographics**	
	Age, year
	Gender
**Traditional CVD risk factors**	
	HDL Cholesterol, mg/dl
	LDL Cholesterol, mg/dl
	BMI, kg/cm2
	Triglyceride, mg/dl
	Diabetes
	Hypertension
	Smoking status
	Cerebrovascular disease
	Coronary artery disease
	Congestive heart failure
	Peripheral vascular disease
	Atrial Fibrillation
**Nontraditional risk factors**	
	Glucose, mg/dl
	hsTNT, ng/l*
	IL-6, ng/l*
	PTH, ng/l
	Anemia
	Alcohol
	Cancer
	COPD
	Connective tissue disorder
	Liver Disease
	Psychiatric Disease
	Albumin, g/dl
	ACR or Urin protein, g/24h
	CRP, mg/l
	eGFR, (ml/min/1·73 m^2^)
	Phosphate, mg/dl
	Uric Acid, mg/dl

*Variables always missing in both datasets.

### 2.3 Design and Setting of CALIBRA Validation Studies

CALIBRA was validated in two independent cohorts, one derived from the FMC NephroCare network and the other derived from the GCKD study. This design allowed testing the accuracy of the model in different clinical settings henceforth enhancing the robustness of our results.

#### 2.3.1 FMC NephroCare Cohort

All patients treated in the FMC NephroCare network from 2017 to 2018 were screened for eligibility. A total of 22,535 subjects who underwent at least 2 visits one year apart and one serum creatinine assessment (s-Cr) during their pre-dialysis care were included. We abstracted all data from the EuCliD^®^ database (Fresenius Medical Care, Deutschland GmbH), as previously described (18, 19).

#### 2.3.2 GCKD Cohort

A second analysis evaluated CALIBRA performance using data of the GCKD cohort (13). Briefly, the GCKD study is an ongoing prospective observational cohort study investigating determinants of kidney disease progression and cardiovascular complications. The GCKD study enrolled 5217 patients with CKD of various etiologies between 2010 and 2012. Patient recruitment and follow-up are organized through a network of academic nephrology centers collaborating with practicing nephrologists throughout Germany. At the time of recruitment, patients were under nephrological care and either had an eGFR of 30–60 mL/min/1.73m2 or overt proteinuria in the presence of an eGFR >60 mL/min/1.73m2. In our validation analysis, only patients with a s-Cr value available at baseline were considered (N=5,159).

In both cohorts, the study endpoint, i.e. cardiovascular hospitalization, was assessed over a 12-month follow up period.

### 2.4 Measures

#### 2.4.1 Endpoint Definition

The primary endpoint was the occurrence of any hospitalization due to CVD events within 1 year after the index date (i.e., the date of the first visit recorded for each patient). The short follow up time was chosen to reflect the relatively high incidence rate of cardiovascular event in NDD-CKD patients. Given that NDD-CKD patients undergo repeated visits during the year, longer prediction horizons may reduce the ability to address changes in risk profiles that merit a timely change in treatment or referral to intensified prevention program. CVD events were defined as the composite of HF, CAD, MI, CRVD, AF, PAD, and sudden cardiac death (SCD). In both validation cohorts, endpoint for CALIBRA validation study was extracted from patients’ chart reports. In particular, concerning patients included in the EuCliD^®^ dataset, endpoints were ascertained by occurrence of suggestive ICD10 codes (**Supplementary Material**; **Appendix 3** (20),) that occurred during a hospitalization. In the GCKD study all endpoints are extracted from patient discharge reports; the process of endpoint adjudication and abstraction in the GCKD study has been described previously (18, 19).

#### 2.4.2 Definition of CKD Stages

Patients were classified according to established GFR categories (21, 22) using eGFR estimates derived by the CKD-EPI equation (23).

#### 2.4.3 Other Input Variables

We assessed demographic, anthropometric, life-style variables at index visit; blood biomarkers were collected and averaged over 12 months before the index date; finally, we abstracted comorbidities and CKD etiologies recorded at the index date.

### 2.5 Statistical Analysis and Model Performance Evaluation

We calculated the incidence density and 95% confidence interval (CI) of CVD events in the study population based on the Poisson distribution.

Since data collected in routine clinical practice are largely incomplete, a suitable prognostic algorithm should be able to handle heavy missingness generation process without loss of accuracy. Since CALIBRA is based on NB networks, no data manipulation was required to explicitly handle missing variables. Therefore, one important objective of the study consisted in assessing the computability and accuracy of equation-based risk scores against CALIBRA.

Model performance was evaluated by concordance statistic and calibration charts. Calibration was visually inspected by plotting the incidence of observed outcomes by quintiles of the risk score. We used the calibration plot to evaluate model discrimination properties across the distribution of the risk score. Overall discrimination was quantified by calculating the area under the receiver operating characteristic curve (ROC AUC) in both validation cohorts.

Furthermore, we investigated CALIBRA non-inferiority relative to scores whose performance has been previously assessed in NDD-CKD patients, including the FHS risk score (24), the INDANA calculator (25), and the ASCVD score of the American College of Cardiology (ACC)/American Heart Association (AHA) guidelines (26). Even though such scores evaluate the risk of cardiovascular events over a longer time horizon, it is important to assess their suitability for clinical practice for a more useful endpoint among NDD-CKD patients. Other risk scores, such as the SCORE2 algorithm have never been validated among CKD-NDD patients and demonstrated a wide range of performance across different European Countries SCORE2 (27). A list of variables included in these tools is provided in **Appendix 4**. Non-inferiority was assessed by checking whether a one-sided confidence interval of the AUC remained entirely above the non-inferiority threshold (σ=0.05). If non-inferiority was achieved, we evaluated whether CALIBRA was superior, defined as ΔAUC>0·05 compared to an existing risk score. Superiority was tested with DeLong non-parametric approach (28). Given the sequential nature of testing in a fixed order method approach, type I error is not inflated by multiple testing. Statistical significance was claimed at α<0.05.

## 3 Results

### 3.1 Sample Characteristics

Baseline demographic and clinical data of the FMC-NephroCare and the GCKD cohorts are reported in **Table 2**. In the EuCliD^®^ dataset, patients were older and had lower eGFR levels. Number of patients lost to follow-up was 7,655 (34%) in the FMC-NephroCare cohort and 337 (6.5%) participants in the GCKD study. CVD hospital admissions were 309 (0.021 events/person*year), and 197 (0.041 events/person*year) in the EuCliD^®^ database and the GCKD cohort, respectively.

**Table 2:** Baseline characteristics of patients from the FMC NephroCare and GCKD cohorts.

Variable	FMC NephroCare Cohort		GCKD Cohort		Effect Size^*^
	N	Mean (SD) or N (%)	N	Mean (SD) or N (%)	
**Age** (year)	22,535	72.15 (11.7)	5,159	60.1 (11.95)	1.03
**BMI** (kg/cm2)	21,655	30.63 (10.92)	5,106	29.84 (5.97)	0.08
**eGFR** (ml/min/1.73 m^2^)	22,535	31.93 (13.4)	5,159	49.23 (18.12)	1.20
**Albumin** (g/dl)	19,004	4.19 (0.4)	5,156	3.83 (0.44)	0.87
**Ferritin** (µg/l)	7,303	222.18 (260.98)	1,315	193.59 (187.85)	0.11
**Haemoglobin** (g/dl)	21,916	12.65 (1.83)	5,052	13.6 (1.67)	0.53
**Phosphate** (mg/dl)	20,362	3.65 (0.74)	5,158	3.43 (0.64)	0.30
**Calcium** (mg/dl)	20,686	9.36 (0.73)	5,159	9.08 (0.62)	0.40
**Sodium** (mmol/l)	20,612	140.17 (3.16)	5,158	139.69 (3.08)	0.15
**PTH (**ng/l)	9,466	131.84 (150.12)	0	.	.
**ACR** (mg/mmol)	90	138.67 (568.28)	5,086	430.34 (960.55)	0.31
**Proteinuria** (g/24h)	8,780	3.58 (150.29)	0	.	.
**Systolic** (mmHg)	17,963	137.33 (18.41)	5,127	139.44 (20.36)	0.11
**CRP** (mg/l)	13,468	9.42 (18.29)	5,157	4.75 (8.35)	0.29
**Glucose** (mg/dl)	19,499	126.45 (48.59)	0	.	.
**HDL Cholesterol** (mg/dl)	7,074	48.3 (16.74)	5,148	51.97 (18.14)	0.21
**LDL Cholesterol** (mg/dl)	7,084	107.59 (219.29)	5,148	118.29 (43.55)	0.06
**Triglyceride** (mg/dl)	15,191	169.84 (113.46)	5,148	199.32 (128.01)	0.25
**hsTNT** (ng/l)	0	.	3,976	17.47 (19.81)	.
**Uric Acid** (mg/dl)	20,273	6.68 (1.61)	5,159	7.2 (1.91)	0.31
**Gender (M)**	22,535	11349 (50.36%)	5,159	3099 (60.07%)	0.67
**Etiology Diabetes**	22,535	3614 (16.04%)	5,159	773 (14.98%)	1.08
**Etiology Polycystic**	22,535	477 (2.12%)	5,159	179 (3.47%)	0.60
**Etiology Hypertension**	22,535	5,281 (23.43%)	5,159	1,187 (23.01%)	1.02
**Etiology Glomerulonephrite**	22,535	987 (4.38%)	5,159	968 (18.76%)	0.20
**Smoking status: ex smoker**	3,502	3,502 (15.54%)	2,223	2,223 (43.09%)	0.24
**Smoking status: no smoker**	10,066	10,066 (44.67%)	2,100	2,100 (40.71%)	1.18
**Smoking status: smoker**	2,274	2274 (10.09%)	821	821 (15.91%)	0.59
**Alcohol: abuse**	8,636	8,636 (38.32%)	977	977 (18.94%)	2.66
**Alcohol: moderate**	0	0 (0%)	4,152	4,152 (80.48%)	.
**Alcohol: abstinence**	6,984	6,984 (30.99%)	0	0 (0%)	.
**Peripheral Vascular Disease**	22,535	1,875 (8.32%)	5,159	487 (9.44%)	0.87
**Coronary Artery Disease**	22,535	4,336 (19.24%)	5,159	1,027 (19.91%)	0.96
**Congestive Heart Failure**	22,535	1,887 (8.37%)	5,159	916 (17.76%)	0.42
**Cerebrovascular Disease**	22,535	1,876 (8.32%)	5,159	505 (9.79%)	0.84
**Connective Tissue Disorder**	22,535	399 (1.77%)	0	0 (0%)	.
**Cancer**	22,535	2,469 (10.96%)	5,159	628 (12.17%)	0.89
**Diabetes**	22,535	9,021 (40.03%)	5,159	1,836 (35.59%)	1.21
**Anemia**	22,535	9,800 (43.49%)	5,159	1,207 (23.4%)	2.52
**Hypertension**	22,535	17,871 (79.3%)	5,159	4,969 (96.32%)	0.15
**Atrial Fibrillation**	22,535	2,337 (10.37%)	5,159	1,046 (20.28%)	0.45
**Diabetes Without Complication (CCI)**	22,535	3,013 (13.37%)	5,159	1,836 (35.59%)	0.28
**Chronic Pulmonary Disease (CCI)**	22,535	1,618 (7.18%)	5,159	352 (6.82%)	1.06
**Psychiatric Disease**	22,535	177 (0.79%)	0	0 (0%)	.
**Liver Disease**	22,535	987 (4.38%)	0	0 (0%)	.
**RRT in 2 years**	9,407	1,817 (19.32%)	4,687	81 (1.73%)	13.60
**RRT in 6 Months**	18,504	801 (4.33%)	4,932	11 (0.22%)	20.52
**CVD Hospitalization in 1 year**	14,880	309 (2.08%)	4,822	197 (4.09%)	0.50
**CKD stage:**					
**1-2**	0	0 (0%)	1,101	1,101 (21.34%)	.
**3**	11,965	11,965 (53.1%)	3,593	3,593 (69.65%)	0.49
**4**	8,026	8,026 (35.62%)	460	460 (8.92%)	5.65
**5**	2,544	2,544 (11.29%)	5	5 (0.12%)	105.92

*Effect size was estimated using Coehn’s d for continuous variables and odds ratio for binary variables.

### 3.2 CALIBRA Discrimination in the FMC-NephroCare Cohort

**Figure 2:**
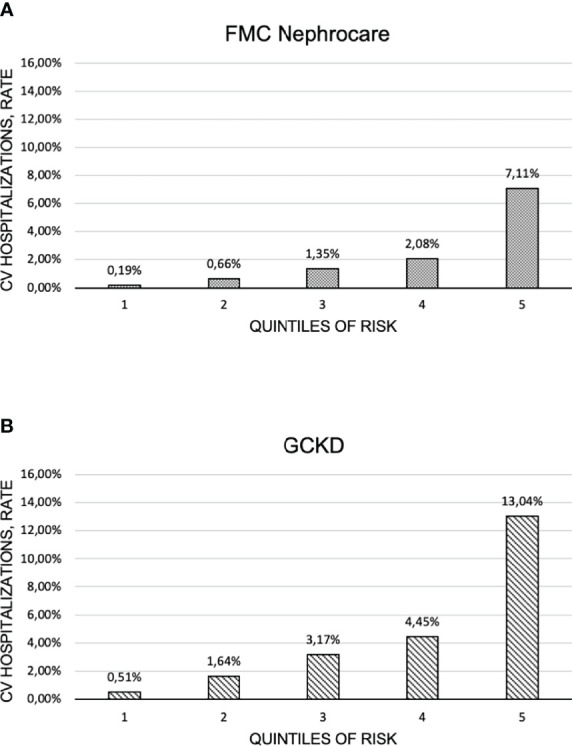
Calibration charts in: **(A)** the FMC Nephrocare and **(B)** the GCKD cohorts.

Our model showed good discriminative ability in the FMC-NephroCare cohort. Concordance statistic was 0.79 (95% CI 0.76-0.81; Stage G3-5). Calibration of predicted versus observed risk is represented in **Figure 2A**. Risk ratios between adjacent quintiles showed that model discrimination was highest at the extreme of predicted risk distribution.

**Table 3** shows performances of CALIBRA and existing CVD risk models. Based on the pre-defined superiority criteria, CALIBRA outperformed FHS (ΔAUC = -0.22 p<0.001), ASCVD (ΔAUC = -0.17, p<0.001) and INDANA (ΔAUC = -0.14, p<0.001).

**Table 3:** Discrimination ability of CALIBRA. Analysis was performed both in the FMC NephroCare cohort and in the GCKD cohort; endpoint horizon: 1 year.

Cohort	Contrast	Effective Sample Size	AUC CALIBRA	AUC benchmark	Delta AUCs	P-value
**FMC Nephrocare**						
** **	**CALIBRA**	14,880	0.79	-	-	-
	**ASCVD - CALIBRA**	3,960	0.76	0.59	**-0.17**	<0.001
	**FHS - CALIBRA**	3,960	0.76	0.54	**-0.22**	<0.001
	**INDANA - CALIBRA**	3,960	0.77	0.63	**-0.14**	<0.001
**G-CKD**						
** **	**CALIBRA**	4,822	0.73	-	-	-
	**ASCVD - CALIBRA**	4,792	0.73	0.61	**-0.12**	<0.001
	**FHS - CALIBRA**	4,792	0.73	0.57	**-0.16**	<0.001
	**INDANA - CALIBRA**	4,792	0.73	0.69	-0.04	<0.001

CALIBRA performance was assessed either including all cases or excluding patients with missing information. The benchmark model scores were computed considering only complete cases. The column “Effective sample size” reports the number of patients included in each analysis. Imputation method: Listwise. Non-inferiority was defined as ΔAUC<0.05, while superiority was set at ΔAUC >=0.05.

### 3.3 CALIBRA Discrimination in the GCKD Cohort

Discrimination in the GCKD study was moderate (AUC=0.73 95% CI 0.70-0.76; GCKD, Stage G1-5). Calibration of predicted versus observed risk is represented in **Figure 2B**. The model better discriminated low and high-risk patients compared to those classified in the central quintiles of risk score distribution.

Based on pre-specified superiority criteria, CALIBRA outperformed FHS (ΔAUC= -0.16, p<0.001), and ASCVD (ΔAUC= -0.12, p<0.001, **Table 3**). Even though statistically significant, the difference in performance between CALIBRA and INDANA did not meet the pre-defined superiority criteria (ΔAUC = -0.04, p<0.001, **Table 3**).

### 3.4 Case Study

To better illustrate the full scope of CALIBRA in facilitating broad prognostic reasoning, we report model metrics generated from a routine clinical examination for a specific patient who presented to an FMC-NephroCare NDD-CKD outpatient clinic (**Supplementary Material**; **Appendix 5**). The patient was 45 years old, male, obese (BMI=41), suffering from stage G4 CKD (eGFR=25), most likely caused by diabetes; he was a past-smoker. Known comorbidities were hypertension and chronic obstructive pulmonary disease (COPD). Blood biomarkers: serum albumin=4.00 g/dl; calcium=8.9 mg/dl; hemoglobin=12.53 g/dl; phosphate=4.19 mg/dl, PTH=289 ng/l, proteinuria=2.49 g/24h.

Based on these data CALIBRA predicted a 14% risk of CVD hospitalization within 1 year.

According to Impact Analysis, proteinuria (normalized likelihood, NL=1.52), diabetes (NL=1.43), COPD (NL=1.35), male sex (NL=1.24), eGFR (NL=1.06), hypertension (NL=1.05), PTH (NL=1.02) increased CVD risk. Conversely, young age was determined as a protective factor (NL=0.35).

VOI metrics were indexed to the highest in rank. For the present case, the greatest reduction in information entropy (i.e. reduction in uncertainty around risk estimated) would be achieved by measuring, in decreasing order of relative importance, C-Reactive Protein (Indexed VOI=0.46), resting hsTNT (Indexed VOI=0.39), and s-Uric Acid (Indexed VOI=0.22).

## 4 Discussion

In this large validation study, CALIBRA consistently showed better discrimination in predicting the risk of NDD-CKD patients to experience a CV hospitalization within 1 year compared to the FHS (24), the ASCVD (26), and INDANA (25) CV risk algorithms.

CALIBRA overcomes inherent limitations of existing CVD risk scores in forecasting CV events among NDD-CKD patients by making use of both traditional and CKD-specific risk factors (i.e. kidney function measures eGFR (10), albuminuria (9, 29) or proteinuria (10, 11), uric acid (9), C-reactive protein (30), among others). To address the lack of comprehensive and sufficiently large dataset for model derivation, we used a novel literature-based Bayesian Network approach by incorporating pooled effect size estimates obtained from published reports of large historical and longitudinal cohort studies; such an approach proved to enhance model discrimination compared to traditional methods. Of interest, one important corollary of literature-based NBCs is their unlimited scalability, in that additional risk factors can be easily added to the model when sufficient evidence would be collected from future studies. As an instance, CALIBRA already includes biomarkers such as hs-cTnT (3, 10) and IL-6 (31) which have been previously shown to provide independent cardiovascular prognostic signals. Unfortunately, these biomarkers were not available in the two study populations, so that their potential incremental discrimination in CALIBRA could not be assessed.

Of importance, CALIBRA superiority against existing cardiovascular risk scores was demonstrated in two independent cohorts, namely the FMC-NephroCare and the GCKD cohorts. These cohorts show substantial differences in their design, geographical area of recruitment, inclusion/exclusion criteria, measurements, and data collection strategies. Indeed, EuCliD^®^ is a medical registry archiving FMC-NephroCare real-world medical data of stage G 3-5 CKD and dialysis patients from multiple countries in Europe, South America, Africa, Middle-East and the Asia-Pacific region with exclusion of Germany. Conversely, the GCKD study is a large prospective observational research that enrolled patients with stages G3, A1-3 or stages G1-2, A3 in Germany (13). In addition, in the EuCliD^®^ registry, medical data were collected by clinicians and nurses as part of their routine activity; biochemical tests, despite automatically uploaded from lab results files, were purposefully prescribed by physicians. On the other hand, in the GCKD cohort, data were collected per protocol at pre-specified assessment timepoints. Consequently, the EuCliD^®^ registry was associated with a higher percentage of missing variables, while the GCKD dataset was almost complete. Overall, our results support the generalizability of CALIBRA across different CKD populations and clinical settings.

Consistent with previous reports (8), our findings showed that the accuracy of FHS in predicting CV events among NDD-CKD patients is poor. Superiority of CALIBRA against the FHS score is not surprising. Indeed, FHS was derived from a general population-based cohort with a low NDD-CKD prevalence; furthermore, it includes traditional risk factors only (24). CALIBRA performance was likewise superior to that of the ASCVD risk prediction tool which considers additional variables including the regular use of medications for diabetes and high blood pressure (26). A third comparison was made relative to INDANA that, in contrast to the other algorithms, includes a measure of kidney function (25). Our findings indicate superior discrimination of CALIBRA compared to that of INDANA. The difference in performance was particularly evident in the FMC-NephroCare cohort. The reduced performance of the INDANA calculator likely depends on its derivation from a randomized controlled trial. In fact, despite this design vouches for the accuracy of data collection in the INDANA study, it may limit the generalizability of the risk score in real life setting where data generation process is driven by clinical decision making and, as a consequence, may lack consistency. On the contrary, CALIBRA was robust to missing variables due to the inherent properties of NB networks. It is worth mentioning that two novel risk models were recently developed to predict CVD risk among CKD patients. In the first study, a novel algorithm incorporatingkidney function and proteinuria to existing risk equations has been derived and validated in a very large collaborative cohort by the CKD Prognosis Consortium (CKD-PC) study group (32). The augmented risk equations showed improved discrimination compared to original risk scores (GFR Patch: AUC=0.75; 95%CI 0.70-0.77; CKD Patch, including both eGFR and ACR: AUC=0.70; 95%CI 0.67-0.73). The second study provides two models for 10-year risk prediction of atherosclerotic CVD, namely a model composed of readily clinically available variables and a biomarker-enriched model (33). Both tools were derived using data from the Chronic Renal Insufficiency Cohort (CRIC) study and showed an AUC of 0.76 (95%CI 0.68-0.85) and 0.77 (95%CI 0.67-0.85), respectively. Of interest, the discrimination ability observed in these studieswas comparable to CALIBRA performance.

Irrespective of discrimination superiority, NB networks have several advantages over equation-based risk scores. First, BNCs account for uncertainty related to variable missingness and provide realistic risk estimation by incorporating distributional assumptions for unmeasured input variables (14). While this property may not provide any apparent advantage in longitudinal cohorts such as the GCKD study given its almost complete datasets, it is particularly useful in real-life applications where traditional, equation-based approaches may not be calculable for many patients with missing information on key risk factors. As expected, the FHS, the INDANA, and the ASCVD scores could not be computed for a large proportion of patients in the FMC-NephroCare cohort due to many parameters not being documented in clinical practice. On the contrary, CALIBRA could be used in all patients and its accuracy remained consistently superior to other risk score.

Finally, NB networks provide additional metrics beyond risk estimations which further support prognostic reasoning in clinical practice. In order to demonstrate the enhanced clinical utility of metrics generated by NBCs, we showed CALIBRA predictions in an exemplary case. Beside accurate CVD risk prediction, NBCs facilitate the assessment of prediction robustness to missing information on risk factors by inspecting VOI. Therefore, VOI metrics may help healthcare professionals prioritize further diagnostic testing by highlighting those that would provide the highest amount of additional prognostic information for the case at hand. Moreover, NBCs rank the impact of observed evidence on each patient’s risk based on NL metrics (16), thus suggesting how individual patients’ risk may be reduced by manipulating modifiable risk factors. This appears particularly relevant for those applications in which changes in lifestyle or medication can be recommended. In fact, the optimal intervention mix may be different for patients with the same overall risk based on the relative contribution of each risk component.

Our study shows some limitations. First, we opted not to include novel markers of glomerular filtration rate, e.g. cystatin C and Beta2-microglobulin (3, 10), into CALIBRA due to their limited availability in the real-world clinical records. Nevertheless, our method for the derivation of Literature-Based NBCs allows to add any further risk factor as soon as sufficient evidence is published and the candidate biomarker is ready to be adopted in clinical practice. Second, CALIBRA shows a slightly reduced discrimination in the GCKD population compared to that achieved in the FMC-NephroCare cohort. This observation could suggest that our model may not fully capture the underlying CVD data generation process in NDD-CKD patients. In fact, CALIBRA was derived using data from both longitudinal and historical cohorts in which endpoint definition, measures, and data collection may be subjected to underreporting of less severe cases. Whereas this potential weakness deserves further investigation, it is important to emphasize that CALIBRA still demonstrated superior accuracy compared to established cardiovascular risk scores even in a rigorous longitudinal cohort study setting.

## 5 Conclusions

In contrast to widely used CVD risk profiles, CALIBRA incorporates both traditional risk factors and CKD-specific prognostic variables to obtain an accurate cardiovascular prediction in non-dialysis dependent CKD patients. Contrary to equation-based scores which may not be calculable for a large proportion of patients with incomplete data, CALIBRA extends to all patients and allows explicit assessment of prediction robustness in case of missing values for key risk factors. Furthermore, CALIBRA can be easily updated by incorporating novel predictors allowing to make full use of the new advancements of research in the field. Our analysis suggests that CALIBRA is a valuable addition to the current tools used by nephrologists to stratify patients’ risk and inform referral to cardiovascular prevention programs.

## Data Availability Statement

The datasets presented in this article were acquired from FMC Nephrocare and GCKD cohorts. The raw clinical data are not readily available because data sharing would violate the terms and conditions under which Fresenius Medical Care acquired the data. Pseudo-anonymized raw data may be accessible under a data sharing agreement upon well-motivated requests by qualified scientists upon approval by the CALIBRA study steering committee.

## Author Contributions

FB and LN contributed to study concept, design, statistical analysis, interpretation of results, manuscript drafting, and approved the final version of the manuscript. CL performed literature search, contributed to interpretation of results, drafted the first version of the manuscript, and approved the final version of the manuscript. JT contributed to interpretation of results and drafted the first version of the manuscript, and approved the final version of the manuscript. JN, MH, MatS, BB, MarS, US, and K-UE contributed to data acquisition, interpretation of results, and reviewed and approved the final version of the manuscript. UT performed literature search, project conceptualization and project administration, and reviewed and approved the final version of the manuscript. CB contributed to study concept, interpretation of results, and approved the final version of the manuscript. CM contributed to interpretation of results, and approved the final version of the manuscript. SoS contributed to interpretation of results and approved the final version of the manuscript. StS contributed to interpretation of results, and reviewed and approved the final version of the manuscript. All authors contributed to the article and approved the submitted version.

## Funding

The GCKD study was/is supported by the German Ministry of Education and Research (Bundesministerium für Bildung und Forschung, FKZ 01ER 0804, 01ER 0818, 01ER 0819, 01ER 0820, and 01ER 0821), KfH Foundation for Preventive Medicine, Innovative Medicines Initiative 2 Joint Undertaking (BEAt-DKD, grant number 115974) and corporate sponsors (www.gckd.org). The funder was not involved in the study design, collection, analysis, interpretation of data, the writing of this article or the decision to submit it for publication.

## Conflict of Interest

LN, JT, FB, SoS, StS, CM, CB, and UT are full time employees at Fresenius Medical Care. CL provided medical writing services on behalf of Fresenius Medical Care. HM reports grants from KfH Foundation of Preventive Medicine, and grants from German ministry of Education and Research. MatS reports grants from Fresenius Medical Care during the conduct of the study. BB reports grants from the Federal Ministry of Education and Research (Bundesministerium für Bildung und Forschung (www.bmbf.de), FKZ 01ER 0804, 01ER 0818, 01ER 0819, 01ER 0820 und 01ER 0821), and grants from Foundation for Preventive Medicine of the KfH (Kuratorium für Heimdialyse und Nierentransplantation e.V.–Stiftung Präventivmedizin; www.kfh-stiftung-praeventivmedizin.de). MarS reports grants from Fresenius Medical Care outside the submitted work. K-UE reports grants from: Astra Zeneca, Bayer, Fresenius Medical Care, Vifor, and Amgen during the conduct of the study, personal fees from Akebia, Astellas, Astra Zeneca, Bayer, and Boehringer Ingelheim, and grants from Genzyme, Shire, and Vifor outside the submitted work.

The remaining authors declare that the research was conducted in the absence of any commercial or financial relationships that could be construed as a potential conflict of interest.

## Publisher’s Note

All claims expressed in this article are solely those of the authors and do not necessarily represent those of their affiliated organizations, or those of the publisher, the editors and the reviewers. Any product that may be evaluated in this article, or claim that may be made by its manufacturer, is not guaranteed or endorsed by the publisher.
