# Supplementary material for: The Cardiovascular Literature-Based Risk Algorithm (CALIBRA): Predicting Cardiovascular Events in Patients With Non-Dialysis Dependent Chronic Kidney Disease
Source: Front Nephrol. 2022 Jul 12;2:922251. doi: 10.3389/fneph.2022.922251 (PMC10479593; doi:10.3389/fneph.2022.922251)
Supplement: Supplementary file 1 [file DataSheet_1.doc]

# Supplementary Material

**The Cardiovascular Literature-Based Risk Algorithm (CALIBRA): predicting cardiovascular events in patients with Non-Dialysis Dependent Chronic Kidney Disease (NDD-CKD).**

[***Appendix 1. List of observational studies concerning the association between cardiovascular hospitalizations and potential risk factors.*** 2](#__RefHeading___Toc71054795)

[***Appendix 2. Process adopted to abstract medical knowledge from the literature and compute model parameters. Details of model derivation were previously described and are protected under an international patent application (1).*** 17](#__RefHeading___Toc71054796)

[***Appendix 3. List of ICD10 codes for Charlson comorbidities (2) considered as cardiovascular endpoint in the EuCliD® dataset.*** 18](#__RefHeading___Toc71054797)

[***Appendix 4. List of variables included in previously published risk scores.*** 19](#__RefHeading___Toc71054798)

[***Appendix 5. CALIBRA output*** 20](#__RefHeading___Toc71054799)

[References 21](#__RefHeading___Toc71054800)

# Appendix 1. List of observational studies concerning the association between cardiovascular hospitalizations and potential risk factors.

|  | **Size** | **First Author** | **Year** | **Definition** | **Source Population** | **URL** |
| --- | --- | --- | --- | --- | --- | --- |
| **Condition** | | | | | | |
| GFR | 637315 | Matsushita | 2015 | Lab | Meta-analysis | https://www.sciencedirect.com/science/article/abs/pii/S2213858715000406 |
| CKD classes | 1120295 | Go | 2004 | Lab | Historical Cohort Study - Kaiser Permanente | https://www.nejm.org/doi/full/10.1056/nejmoa041031 |
| GFR | 148217 | Neri | 2011 | Lab | Ingenix i3 | https://pubmed.ncbi.nlm.nih.gov/21783292/ |
| **Socio-Demographics** | | | | | | |
| Women | 148217 | Neri | 2011 | Administrative Data | Ingenix i3 | https://pubmed.ncbi.nlm.nih.gov/21783292/ |
| Women | 37153 | McCullough | 2007 | Medical Registry | National Kidney Foundation's Kidney Early Evaluation Program | https://jamanetwork.com/journals/jamainternalmedicine/article-abstract/412599 |
| Women | 26147 | Weiner | 2006 | Administrative Data | Pooled 4 Cohort studies (USA) | https://pubmed.ncbi.nlm.nih.gov/16931212/ |
| Age | 148217 | Neri | 2011 | Medical Registry | Ingenix i3 | https://pubmed.ncbi.nlm.nih.gov/21783292/ |
| Age | 26147 | Weiner | 2006 | Self-report | Pooled 4 Cohort studies (USA) | https://pubmed.ncbi.nlm.nih.gov/16931212/ |
| African American | 3785 | Lash | 2016 | Self-report | CRIC Study | https://www.ncbi.nlm.nih.gov/pmc/articles/PMC5295809/ |
| African American | 15010 | Nguyen | 2006 | Self-report | NAHNES III | https://jasn.asnjournals.org/content/17/6/1716.short |
| African American | 26221 | Block | 2013 | Self-report | Da Vita & USRDS | https://cjasn.asnjournals.org/content/8/12/2132 |
| Higher Education | 26147 | Weiner | 2006 | Self-report | Pooled 4 Cohort studies (USA) | https://pubmed.ncbi.nlm.nih.gov/16931212/ |
| **Comorbidities/Complications** | | | | | | |
| Diabetes | 148217 | Neri | 2011 | Diagnostic Codes | Ingenix i3 | https://pubmed.ncbi.nlm.nih.gov/21783292/ |
| Diabetes | 26221 | Block | 2013 | Self-report | Da Vita & USRDS | https://cjasn.asnjournals.org/content/8/12/2132 |
| Diabetes | 2003054 | Wiebe | 2014 | Diagnostic Codes | Alberta Kidney Disease Database | https://www.sciencedirect.com/science/article/abs/pii/S0272638614006969 |
| Diabetes | 439 | Chen | 2013 | Diagnostic Codes | CKD patients - Taiwan | https://journals.plos.org/plosone/article?id=10.1371/journal.pone.0060008 |
| Diabetes | 26147 | Weiner | 2006 | Diagnostic Codes | Pooled 4 Cohort studies (USA) | https://pubmed.ncbi.nlm.nih.gov/16931212/ |
| Diabetes | 1268029 | Tonelli | 2012 | Diagnostic Codes | Alberta Kidney Disease Database | https://www.sciencedirect.com/science/article/abs/pii/S0140673612605728 |
| Diabetes | 113 | Goicochea | 2015 | Medical History | RCT – Allopurinol, CKD | https://www.sciencedirect.com/science/article/abs/pii/S0272638614014760 |
| Diabetes | 37153 | McCullough | 2007 | Medical Registry | National Kidney Foundation's Kidney Early Evaluation Program | https://jamanetwork.com/journals/jamainternalmedicine/article-abstract/412599 |
| Hypertension | 37153 | McCullough | 2007 | Medical Registry | National Kidney Foundation's Kidney Early Evaluation Program | https://jamanetwork.com/journals/jamainternalmedicine/article-abstract/412599 |
| Hypertension | 148217 | Neri | 2011 | Diagnostic Codes | Ingenix i3 | https://pubmed.ncbi.nlm.nih.gov/21783292/ |
| Hypertension | 26147 | Weiner | 2006 | Diagnostic Codes | Pooled 4 Cohort studies (USA) | https://pubmed.ncbi.nlm.nih.gov/16931212/ |
| Hypertension | 2003054 | Wiebe | 2014 | Diagnostic Codes | Alberta Kidney Disease Database | https://www.sciencedirect.com/science/article/abs/pii/S0272638614006969 |
| Psychiatric Conditions | 148217 | Neri | 2011 | Any diagnostic code suggestive of affective disorders or antidepressants/anti-anxiety | Ingenix i3 | https://pubmed.ncbi.nlm.nih.gov/21783292/ |
| Psychiatric Conditions | 15336 | Nitsch | 2011 | GDS scale | UK | https://www.sciencedirect.com/science/article/pii/S0272638610015234 |
| Psychiatric Conditions | 5256 | Lopes | 2002 | Self-Report | DOPPS | https://www.sciencedirect.com/science/article/pii/S0085253815485370 |
| Psychiatric Conditions | 2003054 | Wiebe | 2014 | Diagnostic Codes | Alberta Kidney Disease Database | https://www.sciencedirect.com/science/article/abs/pii/S0272638614006969 |
| Coronary Artery Disease | 2003054 | Wiebe | 2014 | Diagnostic Codes | Alberta Kidney Disease Database | https://www.sciencedirect.com/science/article/abs/pii/S0272638614006969 |
| Coronary Artery Disease | 113 | Goicochea | 2015 | Medical History | RCT – Allopurinol, CKD | https://www.sciencedirect.com/science/article/abs/pii/S0272638614014760 |
| Coronary Artery Disease | 439 | Chen | 2013 | High Risk Category | CKD patients - Taiwan | https://journals.plos.org/plosone/article?id=10.1371/journal.pone.0060008 |
| Heart Failure | 2003054 | Wiebe | 2014 | Diagnostic Codes | Alberta Kidney Disease Database | https://www.sciencedirect.com/science/article/abs/pii/S0272638614006969 |
| Heart Failure | 26221 | Block | 2013 | Diagnostic Codes | Da Vita & USRDS | https://cjasn.asnjournals.org/content/8/12/2132 |
| Heart Failure | 1995 | Trespalacios | 2003 | Diagnostic Codes | USRDS | https://www.sciencedirect.com/science/article/abs/pii/S0272638603003597 |
| Cerebrovascular Diseases | 2003054 | Wiebe | 2014 | Diagnostic Codes | Alberta Kidney Disease Database | https://www.sciencedirect.com/science/article/abs/pii/S0272638614006969 |
| Cerebrovascular Diseases | 26221 | Block | 2013 | Diagnostic Codes | Da Vita & USRDS | https://cjasn.asnjournals.org/content/8/12/2132 |
| Peripheral Artery Disease | 2003054 | Wiebe | 2014 | Diagnostic Codes | Alberta Kidney Disease Database | https://www.sciencedirect.com/science/article/abs/pii/S0272638614006969 |
| Peripheral Artery Disease | 26221 | Block | 2013 | Diagnostic Codes | Da Vita & USRDS | https://cjasn.asnjournals.org/content/8/12/2132 |
| Cardiovascular Disease (Overall) | 148217 | Neri | 2011 | Diagnostic Codes | Ingenix i3 | https://pubmed.ncbi.nlm.nih.gov/21783292/ |
| Cardiovascular Disease (Overall) | 26147 | Weiner | 2006 | Diagnostic Codes | Pooled 4 Cohort studies (USA) | https://pubmed.ncbi.nlm.nih.gov/16931212/ |
| Cardiovascular Disease (Overall) | 1157 | I-Wen Wu | 2013 | Medical History | China | https://pubmed.ncbi.nlm.nih.gov/20119930/ |
| Atrial Fibrillation/Flutter | 132372 | Olesen | 2012 | Diagnostic Codes | Danish Register | https://www.nejm.org/doi/full/10.1056/nejmoa1105594 |
| Autoimmune Diseases | 148217 | Neri | 2011 | Diagnostic Codes | Ingenix i3 | https://pubmed.ncbi.nlm.nih.gov/21783292/ |
| Autoimmune Diseases | 2003054 | Wiebe | 2014 | Diagnostic Codes | Alberta Kidney Disease Database | https://www.sciencedirect.com/science/article/abs/pii/S0272638614006969 |
| COPD | 148217 | Neri | 2011 | Diagnostic Codes | Ingenix i3 | https://pubmed.ncbi.nlm.nih.gov/21783292/ |
| COPD | 26221 | Block | 2013 | Diagnostic Codes | Da Vita & USRDS | https://cjasn.asnjournals.org/content/8/12/2132 |
| Mild Liver Disease | 2003054 | Wiebe | 2014 | Diagnostic Codes | Alberta Kidney Disease Database | https://www.sciencedirect.com/science/article/abs/pii/S0272638614006969 |
| Severe Liver Disease | 2003054 | Wiebe | 2014 | Diagnostic Codes | Alberta Kidney Disease Database | https://www.sciencedirect.com/science/article/abs/pii/S0272638614006969 |
| Cancer, Metastatic | 2003054 | Wiebe | 2014 | Diagnostic Codes | Alberta Kidney Disease Database | https://www.sciencedirect.com/science/article/abs/pii/S0272638614006969 |
| Cancer, Non Metastatic | 2003054 | Wiebe | 2014 | Diagnostic Codes | Alberta Kidney Disease Database | https://www.sciencedirect.com/science/article/abs/pii/S0272638614006969 |
| Cancer | 26221 | Block | 2013 | Diagnostic Codes | Da Vita & USRDS | https://cjasn.asnjournals.org/content/8/12/2132 |
| Smoke | 3938 | Baber | 2013 | Self-Report | REGARDS study | https://www.sciencedirect.com/science/article/abs/pii/S0002870313003499 |
| Smoke | 9270 | Staplin | 2016 | Self-Report | SHARP study | https://www.ncbi.nlm.nih.gov/pmc/articles/PMC4996629/ |
| Smoke | 15336 | Nitsch | 2011 | Self-Report | UK | https://www.sciencedirect.com/science/article/pii/S0272638610015234 |
| Smoke | 26221 | Block | 2013 | Self-report | Da Vita & USRDS | https://cjasn.asnjournals.org/content/8/12/2132 |
| Smoke | 26147 | Weiner | 2006 | Diagnostic Codes | Pooled 4 Cohort studies (USA) | https://pubmed.ncbi.nlm.nih.gov/16931212/ |
| Smoke | 37153 | McCullough | 2007 | Medical Registry | National Kidney Foundation's Kidney Early Evaluation Program | https://jamanetwork.com/journals/jamainternalmedicine/article-abstract/412599 |
| Alcohol drinking | 15336 | Nitsch | 2011 | Self-Report | UK | https://www.sciencedirect.com/science/article/pii/S0272638610015234 |
| Moderate Alcohol drinking | 26147 | Weiner | 2006 | Diagnostic Codes | Pooled 4 Cohort studies (USA) | https://pubmed.ncbi.nlm.nih.gov/16931212/ |
| **Laboratory Test Results** | | | | | | |
| Uric Acid | 148000 | Neri | 2011 | 1 unit increase | Ingenix i3 | https://pubmed.ncbi.nlm.nih.gov/21783292/ |
| Uric Acid | 1157 | I-Wen Wu | 2013 | SUA>7 | China | https://pubmed.ncbi.nlm.nih.gov/20119930/ |
| BMI | 2288 | Ricardo | 2013 | Calculated | Da Vita & USRDS | https://pubmed.ncbi.nlm.nih.gov/18215699/ |
| BMI | 1678 | Weiner | 2008 | Calculated | ARIC study - Stage 3-4 | https://www.ncbi.nlm.nih.gov/pmc/articles/PMC4083633/ |
| BMI | 54506 | Navaneethan | 2016 | Calculated | Cleveland Clinic’s - CKD Registry | https://www.kidney-international.org/article/S0085-2538(15)00070-8/fulltext |
| BMI | 1669 | Elsayed | 2008 | Calculated | ARIC | https://pubmed.ncbi.nlm.nih.gov/18514990/ |
| BMI | 1293362 | Tonelli | 2019 | Calculated | Alberta Kidney Disease Database | https://link.springer.com/article/10.1186/s12882-019-1351-9 |
| BMI | 37153 | McCullough | 2007 | Calculated | National Kidney Foundation's Kidney Early Evaluation Program | https://jamanetwork.com/journals/jamainternalmedicine/article-abstract/412599 |
| HB | 37153 | McCullough | 2007 | Medical Registry | National Kidney Foundation's Kidney Early Evaluation Program | https://jamanetwork.com/journals/jamainternalmedicine/article-abstract/412599 |
| HB | 148000 | Neri | 2011 | Hb<12 | Ingenix i3 | https://pubmed.ncbi.nlm.nih.gov/21783292/ |
| HB | 15336 | Nitsch | 2011 | Quintiles | UK | https://www.sciencedirect.com/science/article/pii/S0272638610015234 |
| HB | 439 | Chen | 2013 | Unit Increase | CKD patients - Taiwan | https://journals.plos.org/plosone/article?id=10.1371/journal.pone.0060008 |
| HB | 980 | I-Wen Wu | 2010 | Unit Increase | Taiwan – AMI Cohort Study | https://pubmed.ncbi.nlm.nih.gov/20119930/ |
| Albumin | 148217 | Neri | 2011 | Alb<3.5 | Ingenix i3 | https://pubmed.ncbi.nlm.nih.gov/21783292/ |
| Albumin | 15336 | Nitsch | 2011 | Quintiles | UK | https://www.sciencedirect.com/science/article/pii/S0272638610015234 |
| Albumin | 1157 | I-Wen Wu | 2013 | Alb<3.5 | China | https://pubmed.ncbi.nlm.nih.gov/20119930/ |
| Albumin | 439 | Chen | 2013 | 1 unit increase | CKD patients - Taiwan | https://journals.plos.org/plosone/article?id=10.1371/journal.pone.0060008 |
| Glycated Hemoglobin | 23296 | Shurraw | 2011 | 1 unit increase | CKD patients - Canada | https://jamanetwork.com/journals/jamainternalmedicine/article-abstract/1106040 |
| Serum Glucose | 148217 | Neri | 2011 | Glu<126 | Ingenix i3 | https://pubmed.ncbi.nlm.nih.gov/21783292/ |
| HDL | 148217 | Neri | 2011 | HDL>60 | Ingenix i3 | https://pubmed.ncbi.nlm.nih.gov/21783292/ |
| HDL | 26147 | Weiner | 2006 | 10 mg increase | Pooled 4 Cohort studies (USA) | https://pubmed.ncbi.nlm.nih.gov/16931212/ |
| LDL | 148217 | Neri | 2011 | LDL>190 | Ingenix i3 | https://pubmed.ncbi.nlm.nih.gov/21783292/ |
| LDL | 26147 | Weiner | 2006 | 20 mg increase | Pooled 4 Cohort studies (USA) | https://pubmed.ncbi.nlm.nih.gov/16931212/ |
| Triglycerides | 148217 | Neri | 2011 | >500 | Ingenix i3 | https://pubmed.ncbi.nlm.nih.gov/21783292/ |
| Microinflammation | 1157 | I-Wen Wu | 2013 | Undefined | Taiwan – AMI Cohort Study | https://pubmed.ncbi.nlm.nih.gov/20119930/ |
| CRP | 697 | Menon | 2005 | CRP>3 mg/L & CRP>6 mg/L | MDRD study | https://pubmed.ncbi.nlm.nih.gov/16014054/ |
| CRP | 3166 | Jalal | 2012 | Hs-CRP>2.1 mg/L | Members of the health insurance company AOK | https://www.ncbi.nlm.nih.gov/pmc/articles/PMC3704176/ |
| CRP | 128 | Goicochea et al | 2008 | 1 mg/dL increase | Spain | https://pubmed.ncbi.nlm.nih.gov/19034331/ |
| CRP | 90 | Goicochea et al | 2012 | 1 mg/dL increase | Spain | https://pubmed.ncbi.nlm.nih.gov/22746155/ |
| IL-6 | 90 | Goicochea et al | 2012 | 2 determinations >6 pg/mL | Spain | https://pubmed.ncbi.nlm.nih.gov/22746155/ |
| TNT | 128 | Goicochea et al | 2008 | 1 unit increase | Spain | https://pubmed.ncbi.nlm.nih.gov/19034331/ |
| hsTNT | 3483 | Bansal | 2015 | Ref: <5 | CRIC | https://pubmed.ncbi.nlm.nih.gov/25278510/ |
| hsTNT | 442 | Hasegawa | 2013 | <9.0 | Japan | https://pubmed.ncbi.nlm.nih.gov/22914904/ |
| Calcium | 327644 | Palmer | 2011 | 1 unit increase | Meta-analysis - CKD Population | https://pubmed.ncbi.nlm.nih.gov/21406649/ |
| Phosphorus | 327644 | Palmer | 2011 | 1 unit increase | Meta-analysis - CKD Population | https://pubmed.ncbi.nlm.nih.gov/21406649/ |
| Phosphorus | 147634 | Wei-Li | 2014 | 1 unit increase | Meta-analysis - Preserved Renal Fuction | https://journals.plos.org/plosone/article?id=10.1371/journal.pone.0102276 |
| Phosphorus | 32608 | Wei-Li | 2014 | 1 unit increase | Meta-analysis | https://link.springer.com/article/10.1007/s10072-014-1850-1 |
| Phosphorus | 10672 | Mehrotra | 2013 | >4.1 | USA | https://www.sciencedirect.com/science/article/pii/S0085253815560535 |
| Phosphorus | 57832 | McGovern | 2013 | >4.6 | UK | https://journals.plos.org/plosone/article?id=10.1371/journal.pone.0074996 |
| Phosphorus | 1157 | I-Wen Wu | 2013 | >4.1 | China | https://pubmed.ncbi.nlm.nih.gov/20119930/ |
| PTH/Ca/Pi Phenotypes | 26221 | Block | 2013 | Cut-off based on guidelines | Da Vita & USRDS11: Dialysis | https://cjasn.asnjournals.org/content/8/12/2132 |
| PTH | 327644 | Palmer | 2011 | 1 unit increase | Meta-analysis | https://pubmed.ncbi.nlm.nih.gov/21406649/ |
| PTH | -- | Van Ballegooijen | 2013 | 1 unit increase | Meta-analysis | https://pubmed.ncbi.nlm.nih.gov/23622902/ |
| PTH | 1157 | I-Wen Wu | 2013 | 1 unit increase | China | https://pubmed.ncbi.nlm.nih.gov/20119930/ |
| BUN | 9420 | Kirtane | 2005 | 1 unit increase | (OPUS-TIMI)-16 - RCT | https://pubmed.ncbi.nlm.nih.gov/15936606/ |
| BUN | 156 | Ostfeld | 2007 | 1 unit increase | Montefiore Medical Center, USA | https://www.einstein.yu.edu/uploadedFiles/EJBM/page3_page7.pdf |
| BUN | 101089 | Smith et al | 2006 | 1 unit increase | Medicare | https://pubmed.ncbi.nlm.nih.gov/16717177/ |
| Proteinuria | 37153 | McCullough | 2007 | Medical Registry | National Kidney Foundation's Kidney Early Evaluation Program | https://jamanetwork.com/journals/jamainternalmedicine/article-abstract/412599 |
| Proteinuria | 266975 | Van Der Velde | 2011 | Reference: ACR<5 mg/g | Meta-analysis | https://pubmed.ncbi.nlm.nih.gov/21307840/ |
| Proteinuria | 920985 | Hemmelgarn | 2010 | Dipstick or ACR | Alberta Kidney Disease Database | https://jamanetwork.com/journals/jama/article-abstract/185313 |
| Proteinuria | 920985 | Bello | 2011 | ACR | Alberta Kidney Disease Database | https://cjasn.asnjournals.org/content/6/6/1418.short |
| Proteinuria | 39405 | Nagata | 2015 | ACR | Meta-analysis | https://academic.oup.com/aje/article/178/1/1/109968 |
| Proteinuria | 3939 | Sandsmark | 2015 | ACR | CRIC Study | https://www.ahajournals.org/doi/full/10.1161/STROKEAHA.115.009861 |
| Proteinuria | 15336 | Nitsch | 2011 | Dipstick | UK | https://www.sciencedirect.com/science/article/pii/S0272638610015234 |

# Appendix 2. Process adopted to abstract medical knowledge from the literature and compute model parameters. Details of model derivation were previously described (1).

The Naïve Bayesian network model is defined by the incidence of the outcome and the conditional probability of each risk factor given the outcome.

From meta-analysis we obtained the following set of information:

1. *pooled adverse outcome incidence I*
2. *pooled risk factor prevalence P_1_, P_2_, …, P_n_*
3. *pooled effect size measures: OR_1_, OR_2_, …, OR_n_*

The conditional probabilities of each risk factors *PRF_1, PRF_2, …, PRF_n* for a given outcome is computed with the following steps:

1. Get I
2. Get *P_1_, P_2_, …, P_n_*
3. Get *OR_1_, OR_2_, …, OR_n_*
4. Compute for each risk factor the contribution to the outcome risk as
   *W_RF_i_ = ln(OR_i_)×*
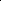
*P_i_*
5. Compute base risk as *λ = -ln((1-I)/I) -*
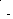
*_i_W_RF_i_*
6. Compute the probability of the outcome given each risk factor as
    *P_ORF_i = exp(*
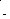
*_j≠i_W_RF_j_+ln(OR_i_)+ λ)/(1+exp(*
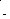
*_j≠i_W_RF_j_+ln(OR_i_)+ λ))*
7. Compute the joint probability of outcome and each risk factor as
    *P_O_RF_i_ = P_ORF_i ×*
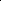
*P_i_* (Bayes’ theorem)
8. Compute the probability of each risk factor given the outcome as
    *PRF_i = P_O_RF_i_ /I*

# Appendix 3. List of ICD10 codes for Charlson comorbidities (2) considered as cardiovascular endpoint in the EuCliD® dataset.

cerebrovascular disease: G45-G46.9, H34.0, I60-I69.9

congestive heart failure: I09.9, I11.0, I13.0, I13.2, I25.5, I42.0, I42.5-I42.9, I43, I50

myocardial infarction: I21-I22.9, I25.2

peripheral vascular disease: I70-I71, I73.1, I73.8, I77.1, I79.0, I79.2, K55.1, K55.8, K55.9, P29.0, Z95.8, Z95.9

# Appendix 4. List of variables included in previously published risk scores.

**Framingham Heart Study (FHS) risk score (3):**

gender, age, total cholesterol, dyslipidemia, systolic blood pressure, antihypertensive, cigarette smoking;

**Atherosclerotic cardiovascular disease risk score (ASCVD) (4):**

gender, age, race, total cholesterol, HDL Cholesterol, diabetes, cigarette smoking, regularly used medications for high blood pressure and diabetes mellitus;

**Individual Data Analysis of Antihypertensive Intervention Trials (INDANA) calculator (5):**

gender, age, serum creatinine, total Cholesterol, height, systolic blood pressure, antihypertensive, diabetes, left ventricular hypertrophy, history of stroke, and history of myocardial infarction, cigarette smoking

# Appendix 5. CALIBRA output


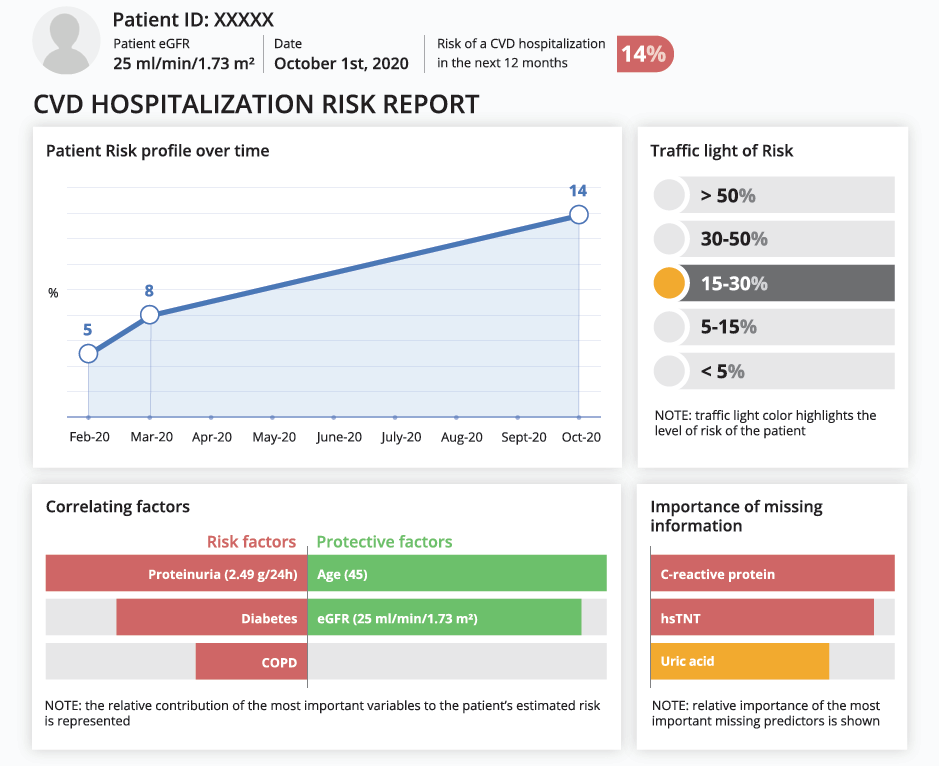


# References

1. Tschulena, U.; Bellocchio, F.; Neri, L.; Amsen, W.; Barbieri C. Medical device and computer-implemented method of predicting risk, occurrence or progression of adverse health conditions in test subjects in subpopulations arbitrarily selected from a totalò population. 2019.
2. Quan H, Sundararajan V, Halfon P, Fong A, Burnand B, Luthi JC, et al. Coding algorithms for defining comorbidities in ICD-9-CM and ICD-10 administrative data. Med Care. 2005
3. D’Agostino RB, Grundy S, Sullivan LM, Wilson P. Validation of the Framingham coronary heart disease prediction scores: Results of a multiple ethnic groups investigation. *J Am Med Assoc* (2001) doi:10.1001/jama.286.2.180
4. Goff DC, Lloyd-Jones DM, Bennett G, Coady S, D’Agostino RB, Gibbons R, Greenland P, Lackland DT, Levy D, O’Donnell CJ, et al. 2013 ACC/AHA guideline on the assessment of cardiovascular risk: A report of the American college of cardiology/American heart association task force on practice guidelines. *J Am Coll Cardiol* (2014) doi:10.1016/j.jacc.2013.11.005
5. Pocock SJ, McCormack V, Gueyffier F, Boutitie F, Fagard RH, Boissel JP. A score for predicting risk of death from cardiovascular disease in adults with raised blood pressure, based on individual patient data from randomised controlled trials. *Br Med J* (2001) doi:10.1136/bmj.323.7304.75
